# Functional restoration of the esophagus after peroral endoscopic myotomy for achalasia

**DOI:** 10.1371/journal.pone.0178414

**Published:** 2017-05-23

**Authors:** Cheal Wung Huh, Young Hoon Youn, Hyunsoo Chung, Yong Chan Lee, Hyojin Park

**Affiliations:** 1 Department of Internal Medicine, Yonsei University College of Medicine, Seoul, Korea; 2 Department of Internal Medicine, Gangnam Severance Hospital, Yonsei University College of Medicine, Seoul, Korea; 3 Department of Internal Medicine, Seoul National University College of Medicine, Seoul, Korea; University Hospital Llandough, UNITED KINGDOM

## Abstract

**Purpose:**

Peroral endoscopic myotomy (POEM) is a new efficacious treatment option for achalasia. We propose to define “esophageal remodeling” as the functional restoration of the esophagus that involves decreased lower esophageal sphincter (LES) pressure, recovery of esophageal body peristalsis, and reduction of luminal diameter. The aim of this study was to investigate “esophageal remodeling” after POEM for achalasia.

**Materials and methods:**

We analyzed data from a prospectively collected database of POEM subjects, which included preoperative and 2-month postoperative Eckardt symptom scores, and results from esophageal high resolution manometry (HRM) and barium esophagogram (BE). We recruited 23 patients (13 male; mean age: 53.9 years) whose preoperative and postoperative HRM and BE results were available, from among 30 patients with achalasia who underwent POEM at two institutions between July 2013 and December 2015.

**Results:**

All patients achieved clinical treatment success (Eckardt score≤3). Partial recovery of esophageal body peristalsis was noted in 1/5 patients with type I (20%), 6/11 with type II (54.5%), and 7/7 with type III (100%) achalasia after POEM. Pan-esophageal pressurization disappeared after POEM in 10/11 type II achalasia patients. The average diameter of the esophageal body after POEM was significantly decreased in all types of achalasia.

**Conclusion:**

POEM provided excellent clinical symptomatic relief and esophageal remodeling in terms of restoration of peristalsis and reduction in diameter of the esophageal body, especially in patients with type III achalasia.

## Introduction

Achalasia is an esophageal motility disorder characterized by impaired deglutitive relaxation of the lower esophageal sphincter (LES) and absence of proper peristalsis of the esophageal body.[1] This physiologic dysfunction leads to the cardinal symptoms of dysphagia, retrosternal pain and regurgitation, and weight loss.[2] Although the exact etiology and pathophysiology of achalasia remain largely unknown, treatments have focused on relaxation or mechanical disruption of the LES to palliate symptoms.

Recently, peroral endoscopic myotomy (POEM) has emerged as a promising and minimally invasive surgical procedure that has demonstrated excellent short-term clinical outcomes.[3–6] Previous studies reported that POEM resulted in not only subjective clinical symptomatic relief but objective improvement of esophagogastric junction (EGJ) pressure measured by manometry.[3–6] In particular, POEM significantly decreased LES resting pressure and integrated relaxation pressure in most cases. [4,7,8]

There have been a few reports about the recovery of esophageal body peristalsis after treatment of achalasia.[8–10] However, whether recovery of esophageal body peristalsis after POEM occurred was not clear. Moreover, studies about the functional restoration of the esophagus in patients with achalasia after POEM are few.[8–11] Therefore, further study is needed to substantiate the recovery of esophageal body peristalsis after POEM. Thus, we hypothesized that POEM provides restoration of esophageal function in terms of peristalsis. In addition, we propose to define “esophageal remodeling” as functional restoration of the esophagus with decreased LES pressure, reduced luminal diameter, and recovery of esophageal body peristalsis. The aim of this study was to investigate “esophageal remodeling” after POEM for treatment of achalasia.

## Methods

### Study subjects

This study was a retrospective review of prospectively collected achalasia data. We recruited 23 patients with achalasia who underwent high resolution manometry (HRM) and barium esophagogram before and after POEM between July 2013 and December 2015 at two tertiary gastroenterology centers (Gangnam Severance Hospital and Severance Hospital, Yonsei University). Achalasia was diagnosed based on clinical symptoms, barium esophagogram, and HRM.

Exclusion criteria were patients with coagulopathy, pregnancy, and patients who rejected manometry or barium esophagogram, or who withdrew informed consent. Symptoms were assessed using the well-established Eckardt symptom scoring system. A postoperative Eckardt score of 3 or less was considered a successful outcome.[12] All patients were followed up with Eckardt symptom score, HRM, and barium esophagogram. This study was approved by the Institutional Review Board at Gangnam Severance Hospital (approval number: 3-2015-0306).

### High-resolution manometry

HRM was performed using the following protocol: a 36-channel, solid-state probe system with high-fidelity circumferential sensors at 1-cm intervals was advanced through the nasal canal (Manoscan; Sierra Scientific Instruments Inc., Los Angeles, CA, USA). Studies were performed with the patient in a sitting position after at least a 6-hour fast. Pressure data of 10 wet swallows were recorded and analyzed by the Manoscan 360. All relevant parameters were calculated according to the Chicago classification v3.0. [13]

All patients were categorized into three subgroups according to the Chicago classification criteria of esophageal motility disorders. Subtype I included patients with a divided mean 4s-integrated relaxation pressure (IRP) ≥15 mm Hg and 100% failed peristalsis. Subtype II patients showed the same features as subtype I patients and had the additional characteristics of pan-esophageal pressurization in at least 20% of swallows. Subtype III patients had the subtype II features and preserved fragments of distal peristalsis or premature (spastic) contractions with at least 20% of swallows.

### Esophagography

Prior to esophagography, patients were prohibited from oral intake for more than 9 hours. Esophagography was performed in the erect anteroposterior projection, left posterior, and anterior oblique projections under fluoroscopy (Shimavision 2000HG; Shimadzu, Kyoto, Japan). Barium sulfate (120 mL) was prepared at a concentration of 140% w/v. The esophageal lumen was observed fluoroscopically during 3 to 4 mouthful swallows at 5-second intervals, and a series of spot images was obtained 1, 2, and 5 minutes after complete swallows. The barium esophagographic studies were reviewed by a gastrointestinal radiologist. Due to the retrospective nature of this study, different levels of magnification and various photographic viewing angles necessitated standardized quantification. Hence, an esophageal width ratio (EWR) was adopted to evaluate the dimensions of the esophageal body by dividing the maximum diameter of the planes perpendicular to the esophageal axis of the barium column by the minimum width of the resting EGJ.[14] Measurement of the maximal caliber of the esophageal body and the width of the resting EGJ was performed on picture archiving and communicating system (PACS) images.

### POEM technique

POEM was performed by Dr. YH Youn and Dr. HS Chung as described by Inoue *et al*. [15] in patients under general anesthesia and CO_2_ insufflation. First, saline supplemented with indigo carmine was injected into the submucosal space on the anterior or posterior wall of the mid-esophagus. Subsequently, a 2-cm longitudinal mucosal incision was made as a mucosal entry into the submucosal space using a triangle-tip knife (KD-640L; Olympus, Tokyo, Japan). Second, the submucosal layer was dissected to create a tunnel along the esophagus and across the EGJ 2 or 3 cm into the proximal stomach. Third, the myotomy was started 2–3 cm below the tunnel entry and extended 2 or 3 cm into the cardia. Lastly, the mucosal entry site was closed with endoscopic clips (EZ-CLIP; Olympus). After POEM, patients received intravenous antibiotics and nutrition for 1–3 days, after which they began to take liquid food that gradually changed to solid food. Patients were followed up with Eckardt scores, HRM, and barium esophagogram 2 months after POEM.

### Statistical analysis

The chi-square test and Fisher’s exact test were used to evaluate associations among various categorical variables, and the *t*-test was used for non-categorical variables in the intergroup comparisons of clinical characteristics. Independent factors related to recovery of esophageal body peristalsis were evaluated by multivariate logistic regression analysis using the score statistic method. The accepted significance level was a *p-*value <0.05. All statistical analyses were performed using SPSS version 18.0 for Windows (SPSS Inc., Chicago, IL, USA) and SAS version 9.2 (SAS Institute, Cary, NC, USA).

## Results

### Patient characteristics

Demographic and clinical characteristics of the study population are shown in Table 1. The average age was 53.9 years (range, 20–84 years), and the ratio of male to female patients was 13:10. Mean duration of symptoms for the 23 patients after achalasia diagnosis was 49.6 months (range 3–324 months). The median Eckardt score before POEM was 6.9 (range 4–11).

**Table 1:** Characteristics of achalasia patients.

Characteristic	Data
Age, mean(range), y	53.9(20–84)
Sex, No. male/female	13/10
Duration of symptom, mean(range), m	49.6(3–324)
Eckardt score, mean(range)	6.9(4–11)
Previous treatments, (*n*)	7
PD	5
BTI	1
HM	1
Achalasis subtypes, (*n*)	
Type I	5
Type II	11
Type III	7

BTI, botulinum toxin injection; HM, Heller myotomy; PD, pneumatic dilation.

Among the 23 patients, 7 patients (30.4%) had received endoscopic or surgical treatments before. Based on the results of HRM, the distribution of achalasia subtype was as follows: type I, 5 patients (21.7%), type II, 11 patients (47.8%), and type III, 7 patients (30.5%).

### Subjective and objective outcome after POEM

Eckardt symptom scores decreased as a result of POEM (pre 6.9 ± 1.7 vs. post 0.6 ± 0.9, *p*<0.001). All of the parameters of lower esophageal sphincter pressure (LESP), 4s integrated relaxation pressure (IRP), distal contractile integral (DCI), and distal latency (DL) between pre- and post-POEM in patients decreased or increased (*p*<0.05). Also, POEM resulted in significantly decreased EWR and diameter of the esophageal body (*p*<0.05) (Table 2).

**Table 2:** Comparison of symptomatic and objective parameters before and after peroral endoscopic myotomy.

Variables	Pre-POEM (mean ± SD) (range)	Post-POEM (mean ± SD) (range)	*P*
Eckardt score	6.9 ± 1.7 (4–11)	0.6 ± 0.9 (0–3)	< .001
HRM parameter			
Resting LESP (mmHg)	36.8 ± 17.7 (12.2–63.5)	14.1 ± 9.5 (2.4–38.2)	< .001
4sIRP (mmHg)	22.1 ± 8.4 (14.0–41.2)	7.4 ± 5.7 (0.3–19.8)	< .001
DCI (mmHg·cm·s)	1079.6 ± 1363.3 (7.4–4336.1)	289.5 ± 379.7 (4.2–1449.2)	.010
DL (s)	3.6 ± 1.2 (1.5–4.2)	6.5 ± 1.4 (4.3–8.7)	< .001
Esophagogram			
EWR	10.1 ± 5.2 (3.2–21.7)	3.4 ± 2.4 (1.1–12.4)	< .001
Diameter of esophageal body (mm)	48.8 ± 29.2 (24.2–166.0)	34.4 ± 22.1 (15.0–111.8)	< .001

DCI, distal contractile integral; DL, distal latency; EWR, esophageal width ratio; IRP, integrated relaxation pressure; LESP, lower esophagus sphincter pressure; POEM, peroral endoscopic myotomy

### Classification of post-POEM esophageal motility patterns

**Fig 1:**
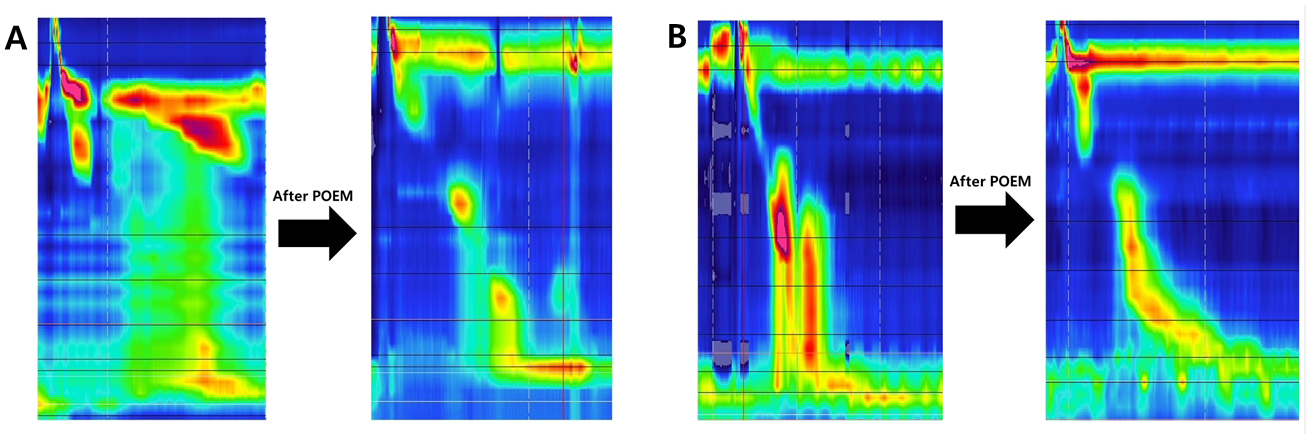
High resolution manometry showing post-POEM recovery of peristalsis. A, Patient with type II achalasia. B, Patient with type III achalasia.

Post-POEM HRM was also interpreted by the Chicago classification v3.0. The new diagnoses of post-POEM esophageal motility patterns are shown in Table 3. According to Chicago classification v3.0, normal esophageal motility is to be defined as normal IRP and >50% effective swallow. Interestingly, pan-esophageal pressurization disappeared after POEM in 10 of 11 patients (90.9%) with type II achalasia (Fig 1A). In addition, all patients (28.6%) with type III achalasia showed partial recovery of esophageal body peristalsis after POEM (Fig 1B). The distal latency was also improved in patients with type III achalasia (pre-POEM, 3.6 ± 1.2; post-POEM, 6.5 ± 1.4) (Table 2).

**Table 3:** New diagnoses of post-peroral endoscopic myotomy(POEM) motility patterns according to pre-POEM achalasia subtype.

Reclassification after POEM	Achalasia subtype prior to POEM, No.of patients (*N* = 23)		
	Type I	Type II	Type III
Type I achalasia	0	1	0
Type III achalasia	0	0	1
Absent contractility	4	4	0
Ineffective esophageal motility	1	6	3
Fragmented peristalsis	0	0	1
Normal esophageal motility	0	0	2

### Factors associated with recovery of esophageal body peristalsis after POEM

After POEM, partial recovery of esophageal body peristalsis was observed in 14 patients (60.8%, 14/23), including one (20%, 1/5) with type I, six (54.5%, 6/11) with type II, and seven (100%, 7/7) with type III achalasia. We analyzed the clinical factors that affected recovery of esophageal body peristalsis after POEM (Table 4). As shown in Table 4, recovery of esophageal body peristalsis after POEM was significantly associated with type III achalasia, short duration of symptoms (<12 months) before POEM, higher resting LES pressure before POEM, and small pre-POEM esophageal body diameter.

**Table 4:** Comparisons of the clinical factors related to recovery of esophageal body peristalsis after peroral endoscopic myotomy.

Variables	Recovery of peristalsis		*P*
	Yes	No	
	(*N* = 14) (*n*,%)	(*N* = 9)(*n*,%)	
Age (years, mean ± SD)	52.0 ± 18.1	54.1 ± 16.9	.782
Sex			.940
Male	8 (57.1)	5 (55.6)	
Female	6 (42.9)	4 (44.4)	
Previous treatments			.242
Yes	3 (23.1)	4 (44.4)	
No	11 (78.6)	5 (55.6)	
Achalasia subtypes			.019
Type I	1 (7.1)	4 (44.4)	
Type II	6 (42.9)	5 (55.6)	
Type III	7 (50.0)	0 (0)	
Duration of symptom (months)			.012
<12	9 (64.3)	1 (11.1)	
≥12	5 (35.7)	8 (88.9)	
Pre-POEM characteristics			
Eckardt score (mean ± SD)	7.1 ± 1.6	6.6 ± 1.8	.432
Resting LESP (mmHg, mean ± SD)	43.9 ± 13.1	21.2 ± 14.1	.001
4s IRP (mmHg, mean ± SD)	24.5 ± 6.1	18.5 ± 10.1	.087
Diameter of esophageal body(mm, mean ± SD)	39.1 ± 10.9	64.6 ± 40.7	.036
Post-POEM characteristics			
Eckardt score (mean ± SD)	0.6 ± 0.8	0.7 ± 1.0	.899
Resting LESP (mmHg, mean ± SD)	17.8 ± 8.6	10.5 ± 11.1	.092
4s IRP (mmHg, mean ± SD)	8.6 ± 5.2	5.4 ± 5.9	.185
Diameter of esophageal body(mm, mean ± SD)	26.5 ±5.7	45.4 ± 30.6	.103

IRP, integrated relaxation pressure; LESP, lower esophagus sphincter pressure; POEM, peroral endoscopic myotomy

However, higher resting LES pressure before POEM and small pre-POEM esophageal body diameter lost their statistical significance upon multivariate analysis. In multivariate logistic regression analysis using the score statistic method, shorter duration of symptoms (<12 months), and achalasia subtype (type III) before POEM were still statistically significant factors associated with recovery of esophageal body peristalsis after POEM (Table 5).

**Table 5:** Multivariate analysis of the clinical factors related to recovery of esophageal body peristalsis after peroral endoscopic myotomy.

Factors		Odds ratio (95% CI)	*P*
Achalasia subtypes	Type I	1	
	Type II	1.943 (0.106–35.596)	.645
	Type III	64.708 (1.098–999.999)	.045
Duration of symptom (months)	≥12	1	
	<12	16.665 (1.303–199.978)	.031

CI, Confidence interval

## Discussion

In this study, we demonstrated that more than half of the patients showed restoration of some intact peristaltic contractions or some remnants of distal esophageal peristalsis in their post-POEM HRM study. The decrease of EWR and maximal diameter of the esophageal body was also observed after POEM. Therefore, these findings propose that POEM provided not only excellent clinical symptomatic relief but also “esophageal remodeling” in terms of restoration of peristalsis and reduced diameter of the esophageal body.

After treatment for achalasia, some patients experienced recovery of esophageal body peristalsis that accompanied the improvement of EGJ manometric profiles. Sharata *et al*. [10] reported that 36% of patients had a return of normal peristalsis (≥70% peristalsis) on post-operative HRM and 47% of patients exhibited partial recovery of peristalsis in another multi-center series of POEM.[8] Nevertheless, it is unlikely that esophageal body peristalsis was consistently affected, which was supported by a recent study that reported only 2 of 66 patients had some degree of antegrade peristalsis after POEM.[9] Therefore, until now, the recovery of peristalsis after POEM was not clear.

In our study, all patients achieved treatment success (Eckardt score ≤3). Partial recovery of esophageal body peristalsis was observed in one patient (20%) with type I, six patients (54.5%) with type II, and seven patients (100%) with type III achalasia after POEM. Pan-esophageal pressurization disappeared after POEM in 10 of 11 type II achalasia patients. In two patients with post-POEM integrated relaxation pressure >15 mmHg, one patient had no peristalsis and the other patient showed premature contractions in postoperative HRM. These two patients also achieved clinical treatment success (Eckardt score ≤3).

Based on these results, the pre-POEM achalasia subtype had some bearing on the pattern found in post-POEM esophageal motility. Despite the small number of subjects, our study demonstrated that patients with type III achalasia were more likely to show recovery of esophageal body peristalsis after POEM. Recovery of esophageal body peristalsis also was significantly associated with shorter duration of symptoms (<12 months), which suggested that progressive stages of the disease process of achalasia are associated with less restoration of peristaltic function. Previous evidence found in a pathological study by Goldblum *et al*. [16] also supports our hypothesis. Those investigators demonstrated that myenteric inflammation with vigorous achalasia (types II and III) was related to a normal number of ganglion cells without neural fibrosis. In contrast, patients with classic achalasia (type I) had few or no ganglion cells with neural fibrosis. They concluded that the earliest pathological changes consisted of myenteric inflammation with injury and subsequent loss of ganglion cells and myenteric nerves. It was suggested that classic achalasia (type I) was a later stage than vigorous achalasia (types II and III) because of progressive myenteric neuron loss. In this regard, recovery of peristalsis after POEM might reflect myenteric plexus inflammation in the distal esophagus, whereas persistent absent peristalsis after POEM might be associated with aganglionosis due to disease progression. In addition, Kim *et al*. [17] recently demonstrated that the majority of esophageal contractions in type III achalasia patients were sequential and associated with adequate clearance of liquid bolus. They also suggested that, rather than a lack of peristalsis, hypertrophy of the muscularis propria resulting in poor distensibility of the esophagus was related to the mechanism of dysphagia in type III achalasia. These characteristics of type III achalasia could explain our finding that type III achalasia patients were more likely to show recovery of esophageal body peristalsis after POEM.

The term “esophageal remodeling” has been used to describe complications regarding food impaction, stricture, and esophageal perforation in eosinophilic esophagitis.[18] Usually, “esophageal remodeling” is used to describe a negative finding (e.g., esophageal fibrosis). In our study, in contrast to the previous definition, we defined “esophageal remodeling” as the functional restoration of the esophagus with decreased LES pressure and lumen diameter as well as recovery of esophageal body peristalsis in achalasia patients.

Our study had several limitations. First, because achalasia is a rare disease, this study was based on a small sample size and only short follow-up of patients at two medical centers. Second, there might be a bias due to the retrospective nature of the current study, although this study was conducted using a prospectively collected database. Third, post-POEM HRM was interpreted by the Chicago classification v3.0, which has limitations in reclassifying esophageal motility after POEM. In addition, we merely evaluated “partial recovery of esophageal body peristalsis” based on changes toward normal-looking contraction in HRM with pressure topography. We did not evaluate bolus clearance after POEM by barium esophagogram or impedance analysis. Therefore, further large studies including bolus clearance are needed to validate our study.

In conclusion, POEM provided not only excellent clinical symptomatic relief, but also “esophageal remodeling” in terms of restoration of peristalsis and reduced diameter of the esophageal body, especially in type III achalasia patients.
