# Correction: Functional restoration of the esophagus after peroral endoscopic myotomy for achalasia

**DOI:** 10.1371/journal.pone.0181289

**Published:** 2017-07-07

**Authors:** Cheal Wung Huh, Young Hoon Youn, Hyunsoo Chung, Yong Chan Lee, Hyojin Park

In the Funding section and the Acknowledgments, the grant number from the funder Yonsei University College of Medicine is listed incorrectly. The correct grant number is: 6-2013-0187.
